# Marital Self-Disclosure Intervention for the Fear of Cancer Recurrence in Chinese Patients With Gastric Cancer: Protocol for a Quasiexperimental Study

**DOI:** 10.2196/55102

**Published:** 2024-04-29

**Authors:** Haiyan Zhao, Ye Zhou, Chong Chin Che, Mei Chan Chong, Yu Zheng, Yuzhu Hou, Canjuan Chen, Yantao Zhu

**Affiliations:** 1 Nursing Department Jingjiang People's Hospital Taizhou China; 2 Chinese Nursing Journals Publishing House Co, Ltd Beijing China; 3 Department of Nursing Science Faculty of Medicine Universiti Malaya Kuala Lumpur Malaysia

**Keywords:** fear of cancer recurrence, dyadic coping ability, gastric cancer, intervention, nursing, protocol, psychological, marital self-disclosure

## Abstract

**Background:**

Patients with gastric cancer experience different degrees of fear of cancer recurrence. The fear of cancer recurrence can cause and worsen many physical and psychological problems. We considered the “intimacy and relationship processes in couples’ psychosocial adaptation” model.

**Objective:**

The study aims to examine the effectiveness of a marital self-disclosure intervention for improving the level of fear of cancer recurrence and the dyadic coping ability among gastric cancer survivors and their spouses.

**Methods:**

This is a quasiexperimental study with a nonequivalent (pretest-posttest) control group design. The study will be conducted at 2 tertiary hospitals in Taizhou City, Jiangsu Province, China. A total of 42 patients with gastric cancer undergoing chemotherapy and their spouses will be recruited from each hospital. Participants from Jingjiang People’s Hospital will be assigned to an experimental group, while participants from Taizhou People’s Hospital will be assigned to a control group. The participants in the experimental group will be involved in 4 phases of the marital self-disclosure (different topics, face-to-face) intervention. Patients will be evaluated at baseline after a diagnosis of gastric cancer and reassessed 2 to 4 months after baseline. The primary outcome is the score of the Fear of Progression Questionnaire-Short Form (FoP-Q-SF) for patients. The secondary outcomes are the scores of the FoP-Q-SF for partners and the Dyadic Coping Inventory.

**Results:**

Research activities began in October 2022. Participant enrollment and data collection began in February 2023 and are expected to be completed in 12 months. The primary results of this study are anticipated to be announced in June 2024.

**Conclusions:**

This study aims to assess a marital self-disclosure intervention for improving the fear of cancer recurrence in Chinese patients with gastric cancer and their spouses. The study is likely to yield desirable positive outcomes as marital self-disclosure is formulated based on evidence and inputs obtained through stakeholder interviews and expert consultation. The study process will be carried out by nurses who have received psychological training, and the quality of the intervention will be strictly controlled.

**Trial Registration:**

ClinicalTrials.gov NCT05606549; https://clinicaltrials.gov/study/NCT05606549

**International Registered Report Identifier (IRRID):**

DERR1-10.2196/55102

## Introduction

In 2020, there were 1,089,103 new cases of gastric cancer and 768,793 deaths worldwide, accounting for 5.6% and 7.7% of the total incidences and deaths, respectively [1]. The standardized morbidity and mortality rates were 11.1/100,000 and 7.7/100,000, respectively, and the cumulative morbidity and mortality risks of those aged 0 to 74 years were 1.31% and 0.90%, respectively. Gastric cancer ranks second in terms of cancer incidence and mortality in China, with 679,000 cases and 498,000 deaths, followed by lung cancer (733,000 deaths) [2]. The disability-adjusted life years associated with gastric cancer in China is 9.825 million person-years, accounting for 44.21% of the gastric cancer cases globally [3].

Advancements in postoperative chemotherapy for gastric cancer have led to an increase in the 5-year survival rate of patients to 68% [4]. Although surgery and combined chemotherapy have improved the survival of patients to a certain extent, the high postoperative recurrence rate and metastatic rate, as adverse psychological events, have a tremendous psychological burden on patients [4]. The fear of cancer recurrence, which first appeared in the subject of oncologic psychology, has particular specificity and independence, which is different from other mental and psychological diseases [5]. Studies reported that about 72% of patients experienced various degrees of fear of cancer recurrence during cancer, of which about 46% were mild or moderate and about 7% were severe [6]. To our knowledge, to date, there have been no psychological intervention studies explicitly targeting the fear of cancer recurrence in Chinese patients with gastric cancer, and only 1 study has conducted a cross-sectional survey of cancer recurrence fear in patients with gastric cancer, which ignored the effect on spouses [7]. However, a spouse’s high fear of cancer recurrence can affect the spouse’s physical and mental health and further jeopardize the patient’s health [8]. Therefore, designing a brief, practical, and feasible intervention to alleviate the fear of cancer recurrence in Chinese patients with gastric cancer and their spouses is critical.

Researchers, patients with cancer, representatives of patient rights, policymakers, and oncology psychologists from many countries reached an expert consensus, which defined the fear of cancer recurrence as “fears and concerns of patients with cancer regarding possible future cancer recurrence and/or metastasis and/or progression” [9]. The primary manifestations of high-level fear of cancer recurrence have been explained as follows: “highly focused, highly concerned, persistent, hypervigilant of physical symptoms and present for at least 3 months” [10].

The treatment cycle of advanced gastric cancer is long, and the recurrence rate is high. Although the progress of medical technology and the resection rate of advanced gastric cancer have increased, the high postoperative recurrence rate and metastatic rate, as adverse life events, have a tremendous psychological impact on patients [11]. Adverse reactions, such as nausea, vomiting, dry mouth, constipation, and bone marrow suppression, aggravate the psychological burden of patients, making them more fearful of the recurrence and progression of the disease [12].

Several studies [12-14] showed that the level of fear of cancer recurrence (as measured by the Fear of Progression Questionnaire-Short Form [FoP-Q-SF]) among patients with gastric cancer (120-170 cases) was middle to high (score of 35.43-42.3 points), the physical health dimension score was 18.09 (SD 4.52), and the social family dimension score was 17.34 (SD 6.34). The scores of the fear of cancer recurrence were higher in patients with gastric cancer than in patients with breast cancer [15] and early-stage prostate cancer [16]. A previous study [17] found that Chinese patients with gastric cancer have less knowledge of the fear of cancer recurrence. High levels of fear of cancer recurrence seriously affect the health of patients with gastric cancer, resulting in a series of dysfunctional behaviors, including avoidance behavior, hypervigilance of symptoms, and inability to plan for the future, which can reduce quality of life, and at the same time, the unmet supportive needs of cancer patients can lead to lower treatment compliance. These effects can persist for months or even years after treatment, and in severe cases, they can lead to anxiety disorders, posttraumatic stress symptoms, and depression [18].

In the relationship intimacy model of couples’ adaptation to cancer by Manne et al [19], the authors emphasized that self-disclosure in relationship-enhancing behaviors is the primary factor affecting the psychological adjustment of cancer patients and their spouses and plays a positive role in the face of cancer. Marriage psychoeducation can help couples understand the value of communicating with each other, provide emotional and problem-focused support, help in the takeover of a spouse’s responsibilities and tasks when one spouse is stressed, and help couples work together to cope when both partners are stressed [20]. In the long run, improvements in dyadic coping are more likely to increase relationship satisfaction than improvements in communication skills [21]. In short, dyadic coping is strongly associated with relationship satisfaction. Therefore, incorporating dyadic coping into a relationship enhancement program may benefit couples’ relationships [22].

Emotional disclosure is a core component of marital emotional support, and it involves expressing feelings, ideas, and opinions to each other [23]. Couple discussion of cancer-related distress may help spouses understand the needs of patients and provide more effective support. Married patients with cancer tend to rate their spouses as their most important confidant. However, patients with cancer often feel constrained in talking about their concerns with their spouses, and partners often withdraw or distance themselves from the emotional distress of patients [24]. These avoidance patterns are present even in couples with satisfying relationships. The inability of patients to talk openly with their spouses about their cancer-related concerns may reduce the ability of couples to cope with stress and compromise the quality of the patient-partner relationship and the patient’s psychological adjustment.

Multiple linear regression analysis results showed that the self-disclosure and intimacy of patients influenced their fear of cancer recurrence [8]. To date, most intervention studies focused on the impact of self-disclosure on the psychological aspect of patients, and they ignored the role of their spouses regarding the psychological state [25-27] and failed to treat patients and their spouses as a whole [12]. Given the vast differences between Western countries and China in terms of race; cultural background; religious beliefs; economic development; and the adaptability and accuracy of the related theory, evaluation scale, and influencing factor analysis, the study results based on Western cultural background cannot be directly applied to the Chinese population. Research on spousal disease communication has primarily focused on patients recovering from breast cancer, lung cancer, and even prostate cancer [28,29]. The treatment cycle for advanced gastric cancer is extended, and the recurrence rate is high, which can intensify the psychological burden on patients [12]. During chemotherapy, patients with gastric cancer encounter numerous dietary restrictions and potential complications, such as nausea and vomiting, heartburn, and acid reflux, resulting in a loss of appetite for food [30]. In Chinese culture, it is believed that food is of utmost importance to people. Being unable to eat or having no appetite is a sign of worsening disease, which further exacerbates both the psychological and physical burdens of patients. No research has been performed on the spousal communication of patients with gastric cancer. Additionally, the research findings of other types of cancers cannot be generalized to patients with gastric cancer, and it is necessary to implement targeted psychological intervention measures for Chinese patients with gastric cancer.

The main objective of this study is to assess a marital self-disclosure intervention that is suitable and feasible based on Chinese culture and clinical practice, focuses on the fear of cancer recurrence, guides patients to adjust to a positive attitude through couple self-disclosure, and improves cancer recurrence fear and the marital relationship with their spouses. If the intervention is proven to be effective, the intervention can be adopted as a practical strategy of psychological care for patients with gastric cancer in China.

## Methods

### Aim

This study aims to develop a marital self-disclosure intervention and evaluate the effectiveness of this intervention for improving the fear of cancer recurrence and the dyadic coping ability in Chinese patients with gastric cancer and their spouses. The trial was registered in November 2022 at ClinicalTrials.gov (NCT05606549).

### Hypotheses

The main hypothesis is as follows: Compared with the control group, participants (patients with gastric cancer and their spouses) in the experimental group show lower scores on the FoP-Q-SF after the intervention.

The secondary hypothesis is as follows: Compared with the control group, participants (patients with gastric cancer and their spouses) in the experimental group show improvements in the dyadic coping ability measured by the Dyadic Coping Inventory (DCI) after the intervention.

### Theoretical Framework

Manne and Badr [31] believed that it is necessary to not only evaluate the psychological adaptation process of patients in the face of cancer, but also evaluate their spouses. On the basis of summarizing multiple theories, such as the resource theory and social cognition theory, Manne proposed the relationship intimacy model of couple adaptation to cancer [32]. This model divides the factors that affect psychological adaptation into 2 parts: relationship-enhancing behaviors and relationship-compromising behaviors. Relationship-enhancing behaviors, including reciprocal self-disclosure, partner responsiveness, and relationship engagement, have a positive effect on intimacy and psychological adaptation, and intimacy also has a positive effect on psychological adaptation. Relationship-compromising behaviors, including avoidance, criticism, and pressure withdrawal, have a negative effect on intimacy and psychological adaptation. In this model, Manne et al [19] emphasized that reciprocal self-disclosure in relationship-enhancing behaviors is the primary factor affecting the psychological adaptation of patients with cancer and their spouses, and plays a positive role in the face of cancer stressors. This model provides a theory basis for this study to analyze the effect of marital self-disclosure on the fear of cancer recurrence and dyadic coping ability from a binary perspective.

### Methodology and Design

A quasiexperimental study with a nonequivalent (pretest-posttest) control group design will be used to examine the effect of marital self-disclosure on the fear of cancer recurrence and dyadic coping ability among patients with gastric cancer and their spouses. The study will be conducted at 2 tertiary hospitals in Taizhou City, Jiangsu Province, China. Considering the time constraints, sample availability, and prevention of contamination, the research will be conducted in the oncology departments (digestive system) of 2 hospitals. The 2 hospitals will be assigned randomly. A total of 42 patients with gastric cancer undergoing chemotherapy and their spouses will be recruited from each hospital. Eligible patients with gastric cancer undergoing chemotherapy who agree to participate and sign the informed consent form will be included in this research. Participants from Jingjiang People’s Hospital will be assigned to the experimental group, while participants from Taizhou People’s Hospital will be assigned to the control group. Patients with gastric cancer share similar characteristics, such as demographics, treatment, and nursing care plans. The treatment methods refer to the “Guidelines for Diagnosis and Treatment of Gastric Cancer” [32]. The chemotherapy scheme is selected according to the patient’s actual situation, which is predicted for 4 to 6 cycles. The study includes 4 stages of face-to-face interventions. The researchers will take measurements at 3 time points: baseline and 2 and 4 months postintervention.

### Study Setting and Recruitment

The study will recruit patients and their spouses who are interested in the study. The nurse practitioners will inform the researchers of the patients’ interest, and the researchers will explain the research methods and requirements, and the ethical information in detail to the patients. Nurse practitioners from the Oncology Department of the 2 hospitals will screen the eligible patients based on the inclusion criteria and will inform the patients and their spouses about the study. The patients who agree to participate will sign a written informed consent form.

### Participants

#### Inclusion Criteria

##### Patients

The inclusion criteria for patients are as follows: (1) the disease meets the diagnostic criteria of the “Guidelines for Diagnosis and Treatment of Gastric Cancer” [32], the preoperative gastroscopy and pathological diagnosis indicate advanced gastric cancer, and the postoperative pathological classification is type II, III, or IV; (2) age ≥18 years and ability to write and communicate effectively; (3) the main caregiver is their spouse; (4) clear consciousness and no understanding barriers; and (5) written informed consent to participate in this study.

##### Spouses

The inclusion criteria for spouses are as follows: (1) age ≥18 years and ability to write and communicate effectively; (2) the main caregiver is the spouse; (3) clear consciousness and no understanding barriers; and (4) informed consent to participate in this study.

#### Exclusion Criteria

##### Patients

The exclusion criteria for patients are as follows: (1) another diagnosis of gastric cancer or another type of cancer, with severe complications, such as gastrointestinal obstruction and perforation; (2) received or is currently receiving psychotherapy from a psychiatrist or psychologist; (3) cognitive impairment or mental impairment; and (4) severe visual, hearing, and speech impairments.

##### Spouses

The exclusion criteria for spouses are as follows: (1) presence of serious physical diseases or acute diseases, such as cancer, stroke, and cardiovascular or cerebrovascular diseases; (2) received or is currently receiving psychotherapy from a psychiatrist or psychologist; (3) cognitive impairment or mental impairment; and (4) severe visual, hearing, and speech impairments.

### Sample Size

The sample size will be calculated using the following formula for sample size based on the study by Charan and Biswas [33]: sample size = 2 (SD) 2 × (Zα/2 + Zβ)2 / D2. It is based on the FoP-Q-SF (primary outcome) from a previously published study [15]. It showed a 95% power to detect a mean difference of 2.88 points between the control and experimental intervention groups in 61 Chinese patients with breast cancer. Statistical significance will be calculated to achieve a statistical power of 0.9 at a 5% significance level for 70 participants in each group. A 2-tailed test will be used to test the hypothesis. The sample size of each dependent variable is 35. To address dropout, an additional 20% is added to the calculation. As a result, the sample size for the experimental and control groups is 42. Consecutive sampling will be used in this study.

### Study Interventions

#### Control Intervention

Participants in the control group will only receive routine nursing care [34]. When patients are admitted to the hospital, apart from routine nursing related to patient treatment, nurses will provide a booklet to patients about gastric cancer and treatment introduction, adverse reactions of chemotherapy, nutrition management, exercise guidance, psychological support (eg, relaxation and music), and life guidance during chemotherapy. In addition, the researchers will contact patients by phone or WeChat every week and send relevant health education messages to reduce the dropout rate. The nurses will not provide patients with any training in communication skills and will not actively encourage couples to discuss cancer-related thoughts and feelings.

#### Experimental Intervention

**Figure 1:**
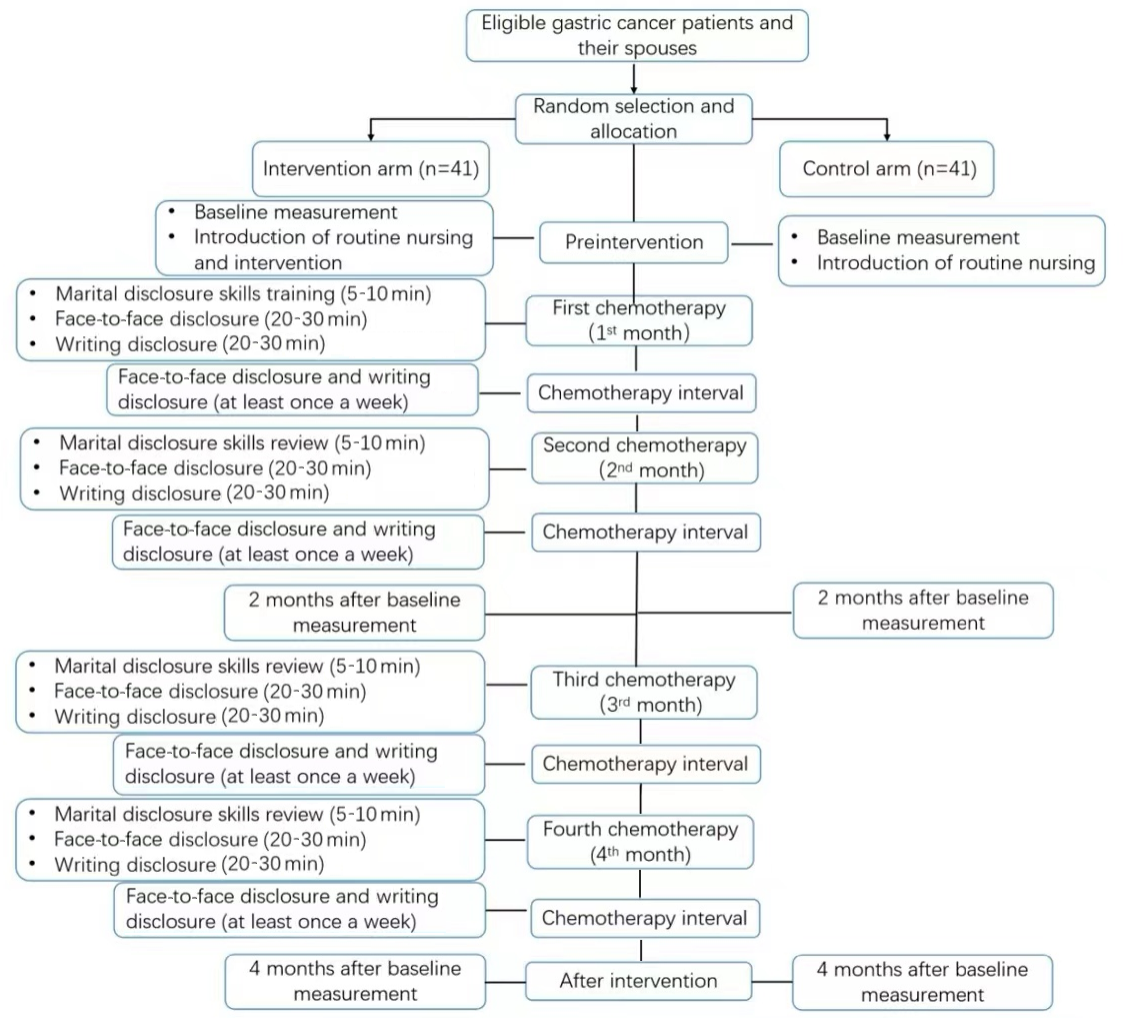
Flowchart of the trial. The primary outcome is the score of the Fear of Progression Questionnaire-Short Form (FoP-Q-SF) for patients. The secondary outcomes are the scores of the FoP-Q-SF for partners and the Dyadic Coping Inventory.

The marital self-disclosure intervention is divided into 4 stages. Each stage will be divided into the chemotherapy and hospitalization phase and the interval and home phase. Both phases include verbal and written disclosures, and the intervention will be conducted in 16 weeks. The flowchart of the trial is shown in Figure 1.

##### Phase 1: Development of the Marital Self-Disclosure Intervention

A previous systematic study [35] by our team found that the most common methods of couple communication are one-on-one and face-to-face communication. They should accept training in marital communication methods before marital self-disclosure, such as how to incorporate the materials from the course into their daily lives through the communication skills learned [36], the strategies for expressing their thoughts and feelings about cancer, and the strategies for accepting and affirming each other’s feelings and opinions [23]. Psychological evaluation indicators are stable, and no significant changes are observed in frequent evaluations [15]. Therefore, 3 to 5 weeks per session can obtain a trend change in the index and will not lead to patient evaluation fatigue, which involves better evaluation frequency. Patients with cancer become unwell due to chemotherapy, and short-term interventions (3-6 stages, 40-60 minutes each step) increase memory and application of what couples have learned and increase patient confidence and motivation to manage their disease [37]. In situations where disclosure to a partner becomes challenging or problematic, individuals may gain some benefit from writing a disclosure diary [15,38,39]. The researchers have developed and designed the intervention. The literature mentions the disclosure needs of cancer couples and the changes in cancer patients’ fear of recurrence during chemotherapy, and provides information on the interview framework, objectives, benefits, mechanisms, and strategies to implement marital self-disclosure. To further improve the marital self-disclosure intervention, the researchers will conduct in-depth interviews with 3 patients with gastric cancer and their spouses to explore the experiences and needs of marital communication. Furthermore, 6 nurses from the oncology ward will be interviewed to explore the intervention preference and obtain their suggestions for the content of marital self-disclosure. The findings of in-depth interviews will be used to improve the frame, stage, time, and disclosure process of marital self-disclosure.

##### Phase 2: Panel Evaluation by Experts

An expert panel will be invited to provide expert inputs for developing the marital self-disclosure intervention. The expert selection criteria are as follows: (1) medical staff specializing in oncology, the gastrointestinal system, and psychology; (2) educational background of a bachelor’s degree or above; (3) deputy senior title in a medical or nursing position or above; (4) rich experience in the above fields and ability to provide professional advice and guidance for this research; (5) working experience of more than 10 years; and (6) willingness to participate in this study. A total of 12 experts will be selected. Before the consultation, the research background and methods will be explained to the experts to obtain support and cooperation.

The contents and methods of marital self-disclosure are derived from the 2 phases mentioned above.

###### Chemotherapy and Hospitalization

The intervention time during hospitalization is the first day after chemotherapy, and the intervention is used once per chemotherapy for a total of 4 times for 45 to 70 minutes each time. The intervention includes verbal and written disclosures. The researcher will preside over the marital self-disclosure process and introduce it to patients with gastric cancer and their spouses (definitions of dyadic coping ability, marital self-disclosure, benefits, stages, process, techniques, and time). Both the patient and their spouse will take turns as speakers and listeners, and receive training or undergo review on marital self-disclosure skills for 5 to 10 minutes each time. The training or review details are shown in Table 1. The training will focus on emphasizing the strengths of dyadic coping and distinguishing between problem-solving and supportive communication. Couples will be encouraged to spend more time understanding each other through supportive communication rather than directly addressing communication issues. Couples will practice marital self-disclosure skills through 4 revealing themes. Couples will be encouraged to express their fear of cancer recurrence. When partners disclose information, supporters should allow the speakers to fully express their feelings and should respond positively to the messages of the speakers, showing empathy for them and acknowledging their points of view.

Each stage has different revealing goals, and the disclosure outline set according to the goals differs. The goals of the personal emotional expression stage are to reveal the person’s thoughts and feelings after the illness, reveal the person’s concerns about the progression of the disease, and express other emotions. The goals of the social cognition expression stage are to disclose the impact of cancer on family or social functioning after the illness, reveal the couple’s specific concerns about the progression of the disease, and express other emotions. The goals of the benefit discovery stage are to disclose the benefits of couples from the experience of illness, reveal positive emotions after the illness, and reveal positive changes that have occurred in their life during treatment. The outlook to the future stage aims to disclose the couple’s plans, reveal the couple’s needs, formulate a cancer recurrence prevention plan, and express other emotions. The patient with gastric cancer and their spouse will disclose information to each other according to the disclosure outline (20-30 minutes) at each stage. When one party is disclosing information, the other party is required to listen actively. After the verbal disclosure, the patient will participate in a written disclosure based on the given topics for 20 to 30 minutes each time. During the written disclosure, the researcher and the patient’s spouse are required to maintain silence and respond to the patient timely if there are any questions. After writing, the researcher will take back the notebook. The disclosure topics of the 4 stages are described in detail in Table 2.

**Table 1:** Disclosure training of the speaker and listener.

Disclosure request	Description
Disclosure request of the speaker	Share a topic-related experience that evokes strong emotions Follow your heart and express your thoughts sincerely Tell your partner about the experience in as much detail as possible, including the event itself and psychological feelings Do not talk too much at one time; pause occasionally to give your partner a chance to show understanding and support
Disclosure request of the listener	Try to stand in your partner’s role and understand their experience Avoid immediately solving the problem or giving advice, focus the conversation on how your partner feels, reveal your thoughts when necessary, and facilitate the expression of your partner Listen reflectively, summarize what your partner has said, pay attention to your tone of voice, maintain eye contact, and nod your head to show understanding

**Table 2:** Marital self-disclosure topics.

Theme and aspects		Description
**First chemotherapy: personal emotional expression**		
	Verbal disclosure goals (couple)	Reveal the person’s thoughts and feelings after the illness Reveal the person’s concerns about the progression of the disease Express other emotions
	Disclosure outline	How have you felt since (your partner) became ill? Are you worried about the progression of the disease? What are the specific aspects? What other emotions did you experience during the treatment?
	Written disclosure (patient)	Please write down your inner thoughts and feelings during the illness and how they affect you
**Second chemotherapy: social cognition expression**		
	Verbal disclosure goals (couple)	Disclose the impact of cancer on family or social functioning after the illness Reveal the couple’s specific concerns about the progression of the disease Express other emotions
	Disclosure outline	What impact does the illness (of your partner) have on your family, work, and social interactions? Are you worried about the progression of the disease? What are the specific aspects? What other emotions did you experience during the treatment?
	Written disclosure (patient)	Please write down the couple’s specific concerns during the illness and how they affect you
**Third chemotherapy: benefit discovery**		
	Verbal disclosure goals (couple)	Disclose the benefits for couples from the experience of the illness Reveal the positive emotions after the illness Reveal the positive changes that have occurred in your life during treatment
	Disclosure outline	Did you benefit from the experience of being sick? What are the specific aspects? What are the good influences on you? What positive emotions did you experience after (your partner) got sick? What positive changes have you made during the (partner) treatment?
	Written disclosure (patient)	Please write down the positive changes and emotions that you have experienced from the illness
**Fourth chemotherapy: outlook to the future**		
	Verbal disclosure goals (couple)	Disclose the future plans of the couple Reveal the needs of the couple and formulate a cancer recurrence prevention plan Express other emotions
	Disclosure outline	What are your plans for the future? What other needs do you have for preventing disease recurrence and what are your plans to prevent the recurrence of the disease? What other emotions did you experience during the treatment?
	Written disclosure (patient)	Please summarize and write about your cancer recurrence prevention plan and hopes for the future

###### Chemotherapy Interval and Homework

With regard to homework requirements, couples will determine the time of disclosure according to the actual situation. The verbal and written disclosures will be conducted at least once a week for 20 to 30 minutes each time. The disclosure outline during the chemotherapy interval is the same as that during the chemotherapy. At the end of every stage, a notebook will be provided to the patient, and the patient will be required to complete homework after chemotherapy.

##### Phase 3: Marital Self-Disclosure Intervention Workshop for Facilitators

First, 2 oncology nurses with strong nursing and communication skills will gain the trust of patients, screen patients who met the inclusion and exclusion criteria, ask them whether they are willing to participate in this study, and assist the consenting patients in signing the informed consent form. Second, participants in the experimental group will receive the marital self-disclosure intervention delivered by 2 communication-led nurses who have master’s degrees in nursing and who are certified psychological counselors. The communication-led nurse will (1) prevent negative interactions; (2) use the checklist to facilitate the disclosures of patients and their spouses; (3) ensure that the communication does not deviate from the outline; and (4) guide the disclosure content to focus on the speaker’s experience. Furthermore, the research nurse will (1) add the WeChat ID of the patient or spouse; (2) provide weekly WeChat reminders; and (3) require the patient or spouse to take photos, upload data, and check in regarding the disclosure execution status and disclosure diary through WeChat. If patients have any nursing or treatment questions, they can consult the research nurse through WeChat. For patients who do not have WeChat, the nurse will follow-up on the implementation status through telephone contact. Moreover, the nurse will record the reasons for patient dropout.

### Instruments and Measures

#### Sociodemographic and Clinical Variables

Variables include the demographic information of patients with gastric cancer and their spouses, including age, gender, education level, employment status, family per capita monthly income, duration of the marriage, and fertility status (couple). In addition, information on clinical variables, including pathological classification, tumor stage, treatment type, and time since cancer diagnosis (patient), will be collected.

#### Fear of Cancer Recurrence for Patients

The FoP-Q-SF is a 1-dimensional scale based on the Fear of Progression Questionnaire (FoP-Q). The FoP-Q-SF for patients includes 12 items, which are rated on a 5-point Likert scale (1 point indicating “never” and 5 points indicating “always”), with a total score ranging from 12 to 60 and with higher scores indicating higher fear of disease progression [40]. Wu et al [41] translated the scale into Chinese and tested its reliability and validity. The authors found that the Cronbach α coefficient of the total scale was 0.886 and the Guttman split-half coefficient was 0.855. Moreover, the Cronbach α coefficients of the 2 extracted common factors were 0.836 and 0.804, respectively, and the Guttman split-half coefficients were 0.806 and 0.828, respectively, which indicated good content consistency and met the requirements of psychometrics.

#### Fear of Cancer Recurrence for Partners

The FoP-Q-SF for partners was developed by Zimmermann et al [42] based on the structure of the FoP-Q-SF scale to assess the degree of partners’ fear of disease progression in patients. The FoP-Q-SF for partners includes 12 items, which are rated on a 5-point Likert scale (1 point indicating “never” and 5 points indicating “always”), with a total score ranging from 12 to 60 and with higher scores indicating higher fear of their spouses’ disease progression. The Cronbach α coefficient of the scale was 0.88. Wu et al [41] translated the scale into Chinese and tested its reliability and validity. The authors found that the Cronbach α coefficient of the total scale was 0.834 and the Guttman split-half coefficient was 0.818. The Cronbach α coefficients of the 2 extracted common factors were 0.835 and 0.699, respectively, and the Guttman split-half coefficients were 0.774 and 0.721, respectively, which indicated good content consistency and met the requirements of psychometrics.

#### Dyadic Coping Ability

The DCI based on the system interaction model was initially developed by Bodenmann [43], which included 6 dimensions (55 items) and used a 5-point Likert scale. After further improvement, the DCI [44] was revised to 37 items and was measured on a 5-point Likert scale ranging from 1 (not at all/very rarely) to 5 (very often). To assess the stress and coping behaviors of couples, the DCI includes 5 subscales for the patient and a corresponding subscale for the partner: (1) stress communication by self; (2) emotion-focused supportive dyadic coping by self; (3) problem-focused supportive dyadic coping by self; (4) delegated dyadic coping by self; and (5) negative dyadic coping by self. The DCI also assesses 2 common dyadic coping behaviors: emotion-focused common dyadic coping and problem-focused common dyadic coping. Xu et al [45] translated this questionnaire into Chinese. The Cronbach α coefficient was 0.73.

### Outcome Assessment

**Figure 2:**
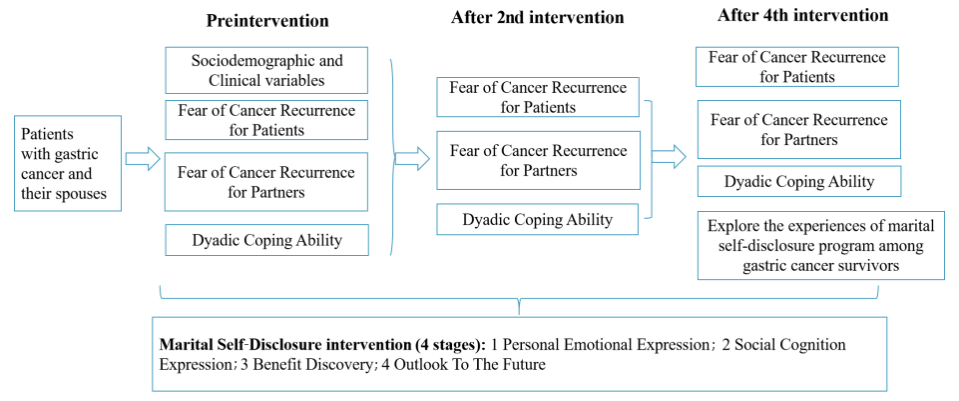
Conceptual framework.

Permission to adopt the 3 questionnaires has been obtained from the original researchers. A trained researcher will assess the outcomes, including sociodemographic variables and psychosocial variables. The sociodemographic variables include demographic and clinical characteristics. The psychosocial variables include fear of cancer recurrence for patients, fear of cancer recurrence for partners, and dyadic coping ability. The psychosocial variables will be repeatedly collected at 3 time points: baseline, after the second session of the intervention (nearly 2 months after baseline), and after the last intervention (almost 4 months after baseline). The control group will be reassessed at the same intervals. Furthermore, participants in the experimental group will be assessed to determine their experiences with the intervention after the last intervention session (Table 3). The conceptual framework is shown in Figure 2.

**Table 3:** Study measures.

Variable	Outcome (measure)	Time point		
		Baseline	2 months after baseline (after the second intervention)	4 months after baseline (after the fourth intervention)
Inclusion criteria	Sociodemographic and clinical information	Yes	No	No
Primary outcome	FoP-Q-SF^a^ for patients	Yes	Yes	Yes
Secondary outcome	FoP-Q-SF for partners and Dyadic Coping Inventory	Yes	Yes	Yes
Intervention experience	In-depth interview	No	No	Yes

^a^FoP-Q-SF: Fear of Progression Questionnaire-Short Form.

### Data Collection and Compensation

Upon agreeing to participate in the study, participants will be required to complete a paper-based baseline survey questionnaire. Participants in the intervention group will have the option to receive the marital self-disclosure intervention directly after completing the survey, while participants in the control group will only receive routine care. Both groups of participants will be tracked for a duration of 16 weeks after recruitment, and data will be collected at 3 time points: baseline and 2 and 4 months postintervention.

To ensure participant confidentiality, information collection will be restricted to face-to-face communication and a paper-based questionnaire. Birth dates or participant addresses will not be collected. All data will be stored on secure internal platforms of Hospitals A and B. At the end of the study period, only researchers will have access to the data set.

At each time point, the research team will contact participants via phone or WeChat, according to their preferences. Participants will be notified of upcoming follow-up studies and will be assisted in completing subsequent survey questionnaires. Upon completing each survey, they will receive small gifts, such as a hand sanitizer. Recruitment is expected to last for 12 months, with an additional 16 weeks needed for follow-up with all recruited participants to complete data collection. Data analysis will commence at the end of recruitment.

### Allocation and Blinding

This study will recruit an equal number of patients with gastric cancer and their spouses. Participants from Jingjiang People’s Hospital will be assigned to the experimental group, while participants from Taizhou People’s Hospital will be assigned to the control group. As the study involves a face-to-face psychological intervention, participants and personnel will be unblinded, but the outcome assessment will be blinded. Participant data will be anonymized using individual participant codes, and the database will not contain participant identifiers (eg, birth dates and case numbers).

### Data Analysis

Statistical analysis will be conducted using IBM SPSS 21.0 (IBM Corp). The level of statistical significance will be set as α=.05. Descriptive statistics will be used to describe the sociodemographic and psychosocial variables, including percentages, means, and standard deviations. For comparing the baseline characteristics of the 2 groups, the Student *t* test or Mann-Whitney *U* test will be used for quantitative variables and the chi-square test will be used for categorical variables. Two-way repeated-measures ANOVA will be performed to determine the presence of a significant change in the fear of cancer recurrence and DCI in the intervention and control groups in order to analyze the effects of the intervention and compare the control group with the experimental group at baseline and 2 and 4 months postintervention. This analysis will be performed on an intention-to-treat basis. A *P*-value of <.05 will be considered statistically significant for differences between the 2 groups.

### Ethical Considerations

The study protocol has been approved by the ethics committees of Jingjiang People’s Hospital and Taizhou People’s Hospital (TZRY-LL-AF/SQ-018-3.0 and KY 2022-167-01). A cover letter will be provided to guarantee human rights and ethical transparency, and inform the participants about the aims and benefits of this study. Participants will be informed that they can voluntarily participate and withdraw from the study without any impact on their treatment or nursing care. If they agree to participate, they will be required to sign an informed consent form with detailed information concerning the goals and procedures of the intervention. The data of this research will be kept confidential and anonymous. The trial was registered in November 2022 at ClinicalTrials.gov (NCT05606549).

## Results

The research activities for this study commenced in October 2022. Participant recruitment and data collection began in February 2023, with the expectation of completion within 12 months (by December 2024). As of October 30, 2023, a total of 97 gastric cancer patients and their spouses have been recruited, and all baseline data have been collected. Once sufficient participants are recruited, subsequent measures will be performed and data analysis will commence. The primary results of this study are anticipated to be announced in June 2024.

## Discussion

### Marital Self-Disclosure Intervention and Protocol Design

Improvement of the fear of cancer recurrence and dyadic coping ability in patients with gastric cancer is necessary. To date, few psychological interventions effectively manage the fear of cancer recurrence in patients with gastric cancer. This is the first and only intervention with the explicit aim of improving the fear of cancer recurrence in Chinese patients with gastric cancer. The intervention focuses on personal emotional expression, social cognition expression, benefit discovery, and outlook to the future, with a further focus on the fear of cancer recurrence. This study will provide evidence of the effectiveness of the marital self-disclosure protocol for improving the fear of cancer recurrence compared with routine nursing care.

There are several advantages of the marital self-disclosure intervention. First, the study involves a long-term intervention that spans from the participant’s diagnosis of gastric cancer until the end of chemotherapy. In a preview study, among patients with gastric cancer in the first, third, and sixth chemotherapy stages, greater family support was associated with a lower fear of cancer recurrence [7]. Therefore, early and lasting intervention is essential.

Second, the study considers the marital factors related to the fear of cancer recurrence and has developed relevant communication topics in each phase. The husband and wife will be trained in marital self-disclosure, and formal disclosure will be made after both husband and wife have mastered the method of disclosure. Moreover, before each phase, the couple will review the method of disclosure again so that the couple can fully grasp the method of disclosure and return it as a homework exercise, making the research results more credible. Finally, this study combines written and verbal representations to facilitate better participant expression of concerns regarding the fear of cancer recurrence.

Third, the study is based on not only a literature review but also Chinese cultural background, clinical practice, and stakeholder input to design a feasible intervention for Chinese patients with gastric cancer.

Fourth, the marital self-disclosure intervention will be designed for implementation by nurses, and it may appeal to nurses who seek flexibility and dynamism in their work. The intervention will train nurses to teach patients and their spouses to use the theory of respect, empathy, and positive attention in emotional communication and marital interaction. Stable, friendly, and trusting relationships will be built between nurses and patients.

### Limitations

This study has some minor limitations. First, this study involves a long-term intervention, and some participants may not complete the study owing to loss of follow-up or physical deterioration during treatment. Second, missing data may influence the results and must be addressed statistically. To overcome this bias, a 20% attrition rate will be included in the sample size calculation, and an intention-to-treat analysis will be performed during the data analysis. Finally, this study will only include patients during chemotherapy.

### Conclusion

This is the first attempt to develop a brief and feasible intervention to manage the fear of cancer recurrence in Chinese patients with gastric cancer. If the marital self-disclosure intervention is effective, it will be implemented at the hospital. This brief and focused intervention will be integrated to enhance clinical practice. Even with null results, participants with gastric cancer and their spouses will obtain more comprehensive self-care nursing knowledge. Furthermore, investigators will obtain a large amount of data to analyze the fear problems of patients with gastric cancer.

## Abbreviations

DCI: Dyadic Coping Inventory
FoP-Q-SF: Fear of Progression Questionnaire-Short Form
